# Prevalence and determinants of hypertension in Myanmar - a nationwide cross-sectional study

**DOI:** 10.1186/s12889-016-3275-7

**Published:** 2016-07-18

**Authors:** Marius B. Bjertness, Aung Soe Htet, Haakon E. Meyer, Maung Maung Than Htike, Ko Ko Zaw, Win Myint Oo, Tint Swe Latt, Lhamo Y. Sherpa, Espen Bjertness

**Affiliations:** Section for Preventive Medicine and Epidemiology, Department of Community Medicine, University of Oslo, Oslo, Norway; International Health Department, Ministry of Health, Nay Pyi Taw, Myanmar; Division of Epidemiology, Norwegian Institute of Public Health, Oslo, Norway; Department of Medical Research, Ministry of Health, Nay Pyi Taw, Myanmar; Department of Preventive and Social Medicine, University of Medicine 1, Yangon, Myanmar; University of Medicine 2, Yangon, Myanmar

**Keywords:** Hypertension, NCD risk factors, Body-mass index, Waist circumference, Smoking, Alcohol, Myanmar, Nationwide

## Abstract

**Background:**

Non-communicable diseases (NCDs), malaria and tuberculosis dominate the disease pattern in Myanmar. Due to urbanization, westernized lifestyle and economic development, it is likely that NCDs such as cerebrovascular disease and ischemic heart disease are on a rise. The leading behavioral- and metabolic NCDs risk factors are tobacco smoke, dietary risks and alcohol use, and high blood pressure and body mass index, respectively. The study aimed at estimating the prevalence and determinants of hypertension, including metabolic-, behavioral- and socio-demographic risk factors.

**Methods:**

A nationwide, cross-sectional study of 7429 citizens of Myanmar aged 15–64 years were examined in 2009, using the WHO STEPS methodology. In separate analyses by gender, odds radios (ORs) and 95 % confidence intervals (CIs) for determinants of hypertension were estimated using logistic regression analyses. Confounders included in analyses were chosen based on Directed acyclic graphs (DAGs).

**Results:**

The prevalence of hypertension was 30.1 % (95 % CI: 28.4–31.8) in males and 29.8 % (28.5–31.1) in females. The mean BMI was 21.7 (SD 4.3) kg/m^2^ for males and 23.0 (5.1) kg/m^2^ for females. In fully adjusted analyses, we found in both genders increased OR for hypertension if the participants had high BMI (males: OR = 2.6; 95 % CI 2.1–3.3, females: OR = 2.3; 2.0–2.7) and high waist circumference (males: OR = 3.4; 1.8–6.8, females: OR = 2.7; 2.2–3.3). In both sexes, associations were also found between hypertension and low physical activity at work, or living in urban areas or the delta region. Being underweight and use of sesame oil in cooking was associated with lower odds for hypertension.

**Conclusions:**

The prevalence of hypertension was high and associated with metabolic-, behavioral- and socio-demographic factors. Due to expected rapid economic growth in Myanmar we recommend similar studies in the future to follow up and describe trends in the risk factors, especially modifiable factors, which will most likely be on rise. Studies on effectiveness on interventions are needed, and policies to reduce the burden of NCD risk factors should be implemented if proven effective in similar settings.

## Background

Non-communicable diseases (NCDs) and malaria, tuberculosis and lower respiratory infections dominate the disease pattern in Myanmar, along with tobacco smoking, high systolic blood pressure and dietary risks as the top three NCD risk factors [1]. Due to urbanization, more westernized lifestyle and economic development in Myanmar, it is likely that there will be an increase in daily smokers, alcohol consumption, unhealthy food habits, physical inactivity, obesity, hypertension and NCDs such as cerebrovascular disease and ischemic heart disease [2, 3]. This kind of health transition has previously been described in the Asian region in countries under rapid economic development [3].

Socioeconomic improvements, welfare policy and improved public health services are drivers of a country’s health transition, which is composed by a demographic transition and an epidemiologic disease transition. Myanmar is currently implementing several reforms aiming at improved living conditions of its population. Concomitant, foreign countries are making investments, which along with the reforms are leading to economic growth and further development [1, 4]. When reaching ordinary people, both will probably impact on the disease pattern and life expectancy, leading to a reduction in infectious diseases and an increase in non-communicable diseases (NCDs). However, similar to many other countries it is likely that development, and thus the disease and risk factor pattern, will differ across urban-rural- and ethnic gradients [3]. It is already documented that health disparities follow these gradients in Myanmar [5]. About 70 % of the 50 million population of Myanmar is currently living in rural areas, and there are more than 100 different ethnic groups with Bamar as the dominant one constituting two-thirds of the total population. Several of the ethnic groups have distinct cultural hallmarks, and main parts of their population living in distinct geographical areas, states or regions.

The long coastline and the fertile land along the many rivers characterize the geography, and could ensure access to seafood and agricultural products for a large proportion of the population. Even in the dry area and the mountain area, the rivers provide supply of fish. However, in the border areas to the east, north and north-west, the access to food is more limited. The differences in access to food across Myanmar is reflected in high frequencies of stunting and underweight in some parts of the country [6]. One fourth of the population lives below the poverty limit [7], and life expectancy at birth is 63.6 years for males and 68.5 years for females [8, 9]. The governmental health expenditures was among the lowest in the world, only 0.89 % of Gross Domestic Product in the financial year 2013/2014 [4].

Although a recent systematic review and meta-analysis regarding prevalence, awareness and control of hypertension in Myanmar was published [10], only a limited number of studies have been conducted.

In the present cross-sectional study of 15–64 year old citizens of Myanmar who were examined in 2009, the objectives were to estimate the prevalence of hypertension, and the associations with selected metabolic-, behavioral- and socio-demographic determinants of hypertension. Findings will serve as baseline information, give clues to targets for interventions, and compared to future data on NCD risk factors, trends can be estimated, as well as projections of the burden of NCDs.

## Methods

We analyzed original data from the Non-communicable Disease Risk Factor Survey Myanmar 2009. Further details on population and methods, selected descriptive results and questionnaire can be found elsewhere [11].

### Population

The inclusion criteria for the study population were ages of 15–64 years, both genders, and all ethnic groups. Mentally and physically too ill subjects and retarded patients, temporary residents (<6 months), and armed forces personnel, prisoners, hospitalized patients, monks, and nuns, were not invited to participate.

The sample size was calculated using the formula *N* = z^2^ P (1-P) / e^2^ where z = α error (5 %); *P* = prevalence of major NCD risk factors (50 %); e = precision (5 %) [11]. Thus, *N* = (1.96)^2^*(0.5)*(1–0.5)/0.05^2^ = 384. Given subgroup analyses of 10 groups (5 age groups and 2 sex groups or urban-rural groups), a cluster sampling design (described below) with a design effect set at 1.5, and 20 % non-response, the sample size was estimated to be 7200 (*N* = 384*(5*2)*1.5/(1–0.2)) [11].

The sampling procedure was the multistage cluster sampling method, with self-weighting sampling procedures. In the first stage, a total of 50 townships from the four regions (delta, plain, hilly and coastal) of Myanmar were selected using the probability proportionate to population size method (PPS). Then, in the second stage, two wards (urban part of township) and three villages (rural part of township) were chosen from each township, using the PPS method, making a total of 100 wards and 150 villages as secondary sampling units (SSUs). In the third stage, 22 households were selected randomly from each ward, and 35 households selected randomly from each village. In stage two and three, the distribution of the households selected from each township followed the urban-rural distribution of the population in Myanmar, which is about 30:70 [12]. Finally, one person from each household who met the inclusion criteria was selected using the Kish table. To summarize, there were 2200 study participants from urban areas, i.e. the wards (100 wards x 22 households), and 5250 participants from rural areas, i.e. the villages (150 villages x 35 households), making a total of 7450 participants. Data was cleaned, and outliers with values outside the range set by the WHO STEPwise approach to Surveillance (STEPS) [13] were excluded giving a total of 7429 participants. After exclusion of 110 pregnant women, the final dataset included 7319 (98.2 %) respondents, and analyses were performed in accordance with the WHO STEPS guideline.

### Methods

We used data from a household-based cross-sectional survey, using the WHO STEPS methodology [13]. STEPS is a standardized method for collecting and processing data, and covers three different levels of assessment. Step 1 is a questionnaire, step 2 requires physical measurements, and step 3 consists of biochemical measurements.

Our data include the core and expanded indicators of both Step 1 and Step 2. The core items contains questions needed to calculate basic variables, for example “do you currently smoke any tobacco products”, while the expanded items asks for more detailed information, like smoke history in the past and number of cigarettes smoked every day [14]. The WHO STEPS Instrument questionnaire was translated into local language for the different areas in Myanmar and the information was collected by face-to-face interviews. Data were collected between May and July 2009, and the physical measurements included height, weight, blood pressure, heart rate, and waist and hip circumferences. Research assistants were trained and followed-up to assure the quality and validity of the measurements. Supervisors assessed the completeness and consistency of the questionnaire after each interview. Measuring tape was used to measure individual’s body height to the nearest to 0.1 cm, without foot wear and any head gear. Body weight was measured with a portable electronic weighing scale to the nearest 0.1 kg. The participants were requested to wear light clothes without footwear during weighing. Waist and hip circumference were measured to the nearest to 0.1 cm, using measuring tape. Waist circumference was taken at midpoint between the lower margin of the last palpable rib and the top of the iliac crest (hip bone), in the standing position without clothing and directly over the skin, according to the WHO guideline. The hip circumference was measured to the nearest to 0.1 cm at maximum circumference over buttocks horizontally. Body Mass Index (BMI) was calculated as weight in kilograms divided by the height in meters squared.

### Variables

After the questionnaire was filled in, the participants rested 30 min before sitting blood pressure was measured three times using the automatic sphygmomanometer “OMRON”. The mean of the two last measurements was used for all analyses. Hypertension was defined as systolic blood pressure of 140 mmHg and greater or diastolic blood pressure of 90 mmHg or greater, or currently taking antihypertensive medications in accordance with WHO criteria [13].

BMI was operationalized into underweight (<18.50 kg/m^2^), normal weight (18.50–24.99 kg/m^2^) and overweight (≥25.00 kg/m^2^) following WHO criteria [15]. Waist circumference (WC) cut-off points were 102 cm or greater for men and 88 cm or greater for women according to WHO criteria [16]. Vigorous activity was defined by asking if the participants experienced large increase in breathing or heart rate for at least 10 min continuously. The STEPS survey did only include questions regarding vigorous activity at work or in recreation time, not during travel time.

Smoking and use of smokeless tobacco were operationalized into groups consisting of those who never had smoked tobacco or used smokeless tobacco, those who formerly used it but have quit, and those who currently is using it. Alcohol was operationalized into non-users and drinkers-group.

WHO recommends a minimum of 400 g of fruit and vegetables per day [13], which is about five daily servings. Therefore, daily fruit and vegetable use were operationalized into two groups with five or more serving per day, and less than five servings per day. Oil use was operationalized into groups depending on which type of oil most often were used for meal preparation - peanut oil, palm oil, mix of peanut and palm oil, sesame oil, or other oils.

The regions were operationalized into the four groups: central plains, hilly, coastal and delta region. Villages were selected for rural areas and wards were selected to represent the urban population. Ethnicity was operationalized in two groups, the main ethnic group Bamar and all the others.

Years at school were operationalized into four groups following the school system in Myanmar. The groups were no school, 1–5 years at school (5 years are compulsory), 6–11 years at school, and more than 11 years at school (higher education). Annual income was defined as total earnings for the household. Marital status were operationalized into those who were currently married, those who never have been married, and those who were separated, divorced or widowed.

### Statistical analysis

Odds ratio (OR) and 95 % confidence intervals (CIs) for determinants of hypertension was estimated using logistic regression analyses. Analyses were done separately for male and females. We identified confounders for each of the association-outcome-relationships drawing Directed Acyclic Graphs (DAGs) [17, 18]. Based on the DAG, for the associations between sociodemographic variables and hypertension all variables in Table 3 were included in analyses as confounders (i.e. age, urban/rural, region, years at school, ethnicity). For the associations between metabolic determinants (Body mass index and Waist circumference) and hypertension (Table 4), we adjusted for sociodemographic confounders (age, urban/rural, region, and years at school) and behavioral confounders (smoking status, smokeless tobacco status, daily fruit and veg. use, oil use, current alcohol drinkers, and vigorous activity at work). For the associations between behavioral determinants (smoking status, smokeless tobacco status, daily fruit and veg. use, oil use, current alcohol drinkers, and vigorous activity at work) and hypertension we adjusted for socio demographic confounders (age, urban/rural, region, years at school). Potential interaction between sex and selected determinants on hypertension was taken care of by conducting separate analyses for males and females. No interaction terms were included in analyses.

Prevalence estimates of hypertension were adjusted to the WHO world standard population [14]. Level of statistical significance was set to *p* ≤ 0.05 or 95 % CI. Data was analyzed using the SPSS version 22 and Stata/IC 14.

## Results

The prevalence of hypertension was 30 % for both sexes, and 6 % males and 11 % females of the total population used anti-hypertensive medications (Table 1). Mean systolic blood pressure was 5 mmHg higher for males than for females, while there was no difference in mean diastolic blood pressure. Mean BMI was low for both sexes, 21.7 kg/m^2^ for males and 23.0 kg/m^2^ for females. The prevalence of smoking, alcohol drinking and physical activity was considerable higher among males than females (Table 1).

**Table 1:** Sociodemographic and CVD risk factors among 15–64 year old citizens of Myanmar, by gender and for the total population

	Male	Female	Total
	*n* = 2862	*n* = 4457	*N* = 7319
Age, mean (SD) years	40.3 (13.4)	40.4 (12.8)	40.4 (13.0)
Systolic blood pressure, mmHg, mean (SD)	130.1 (19.6)	125.4 (20.8)	127.5 (20.5)
Diastolic blood pressure, mmHg, mean (SD)	80.4 (13.0)	79.6 (12.2)	79.9 (12.5)
Hypertension^a^, %	30.1	29.8	29.9
Using antihypertensives^b^, %	5.5	10.7	8.7
Annual income per household, MMK^c^, median (interquartile range)	1000000 (600000–1800000)	842500 (520000–1456000)	960000 (541632–1560000)
Years at school, mean (SD)	7.7 (4.0)	6.7 (4.2)	7.1 (4.1)
BMI, kg/m2, mean (SD)	21.7 (4.3)	23.0 (5.1)	22.5 (4.9)
Waist circumference, cm, mean (SD)	76.3 (10.6)	75.7 (11.2)	75.9 (11.0)
Rural, %	70.2	70.5	70.4
Region, %			
Central plains	38.4	31.8	34.3
Hilly	18.4	13.8	15.6
Coastal	11.7	15.3	13.9
Delta	31.5	39.1	36.1
Ethnicity Bamar, %	73.6	72.9	73.1
Marital status, %			
Currently married	73.1	67.0	21.9
Never married	23.1	21.2	69.3
Sep/Div/Wid	3.7	11.8	8.7
Currently smoking, %	44.8	7.9	22.3
Currently using smokeless tobacco, %	51.4	16.4	30.1
≥5 servings of fruit and veg. daily, %	11.0	9.8	10.2
Peanut oil use, %	41.8	42.6	42.3
Palm oil use, %	19.1	21.1	20.3
Mixed Peanut and palm oil use, %	11.7	11.1	11.3
Sesame oil use, %	18.8	17.0	17.4
Other oil use, %	9.0	8.6	8.7
Current alcohol drinkers, %	31.4	1.6	13.2
Vigorous activity at work, %	41.1	15.0	25.2
Vigorous activity in recreation time, %	12.7	2.0	6.2

^a^SBP ≥ 140 or DBP ≥ 90, or uses antihypertensive medications; ^b^Percentage who uses antihypertensives by the entire population; ^c^1 USD = 1.280 MMK, 26. November 2015

The prevalence of hypertension increase for both genders from lowest age group (15–24 years: male: 12.9 %; female 7.5 %) to highest age group (55–64 years: male: 50.1 %; female: 53.4 %) (Table 2). Adjusted to the WHO world population the prevalences for all ages (total) were 26.5 % (95%CI 24.9–28.2) for males and 25.2 % (23.9–26.4) for females.

**Table 2:** Prevalence of hypertension among 15–64 year old citizens of Myanmar, by age group and adjusted^a^ to the WHO world standard population

Age group		Male		Female		Total
	n	% (95 % CI)	n	% (95 % CI)	N	% (95 % CI)
15–24	412	12.9 (10.0–16.5)	576	7.5 (5.6–9.9)	988	9.7 (8.0–11.7)
25–34	618	18.4 (15.6–21.7)	934	15.5 (13.3–18.0)	1552	16.7 (14.9–18.6)
35–44	650	29.8 (26.4–33.5)	1135	28.0 (25.5–30.7)	1785	28.7 (26.6–30.8)
45–54	649	36.5 (32.9–40.3)	1040	40.3 (37.3–43.3)	1689	38.8 (36.5–41.2)
55–64	513	50.1 (45.8–54.4)	732	53.4 (49.8–57.0)	1245	52.0 (49.3–54.8)
Total	2842	30.1 (28.4–31.8)	4417	29.8 (28.5–31.1)	7259	29.9 (28.9–31.0)
Total adj^a^		26.5 (24.9–28.2)		25.2 (23.9–26.4)		25.7 (24.7–26.7)

^a^ adjusted to the WHO world standard population

Table 3 presents associations (ORs) between selected socio-demographic factors with hypertension, in Model 1 adjusted for age, and in Model 2 adjusted for all variables in table. In both males and females, we found that higher age, urban living and living in Delta area was significantly associated with increased odds for hypertension, while years at school and ethnicity were not (Table 3, Model 2).

**Table 3:** Prevalence of hypertension by gender and by sociodemographic determinants and their associations (Odds Ratio (OR)) with hypertension among 15–64 year old citizens of Myanmar

	Male						Female					
	N	No. (%) with hypertension^a^	Model 1		Model 2		N	No. (%) with hypertension^a^	Model 1		Model 2	
	2862		OR (95 % CI) ^b^	*p*-value	OR (95 % CI) ^c^	*p*-value	4457		OR (95 % CI) ^b^	*p*-value	OR (95 % CI) ^c^	*p*-value
Age												
15–24	415	53 (12.9)	1.00 (ref.)		1.00 (ref.)		518	43 (7.5)	1.00 (ref.)		1.00 (ref.)	
25–34	619	114 (18.4)	1.53 (1.07–2.18)	0.018	1.59 (1.11–2.27)	0.01	945	145 (15.5)	2.28 (1.59–3.26)	<0.001	2.3 (1.60–3.29)	<0.001
35–44	657	194 (29.8)	2.88 (2.06–4.02)	<0.001	2.91 (2.08–4.08)	<0.001	1144	318 (28.0)	4.82 (3.45–6.76)	<0.001	4.91 (3.5–6.89)	<0.001
45–54	656	237 (36.5)	3.90 (2.80–5.42)	<0.001	3.94 (2.82–5.52)	<0.001	1049	419 (40.3)	8.36 (5.99–11.69)	<0.001	8.52 (6.06–11.98)	<0.001
55–64	515	257 (50.1)	6.80 (4.86–9.52)	<0.001	6.86 (4.85–9.7)	<0.001	738	391 (53.4)	14.21 (10.09–20.0)	<0.001	14.63 (10.28–20.82)	<0.001
Urban/rural												
Urban	851	299 (35.3)	1.00 (ref.)		1.00 (ref.)		1311	438 (33.6)	1.00 (ref.)		1.00 (ref.)	
Rural	2011	556 (27.9)	0.71 (0.59–0.85)	<0.001	0.71 (0.59–0.85)	<0.001	3146	878 (28.2)	0.81 (0.70–0.94)	0.004	0.81 (0.70–0.95)	0.008
Region												
Central Plains	1093	294 (27.1)	1.00 (ref.)		1.00 (ref.)		1410	400 (28.6)	1.00 (ref.)		1.00 (ref.)	
Hilly	534	147 (27.63)	1.26 (0.99–1.61)	0.062	1.21 (0.9–1.64)	0.198	631	183 (29.2)	1.21 (0.97–1.51)	0.062	1.09 (0.83–1.43)	0.547
Coastal	333	93 (28.62)	1.15 (0.86–1.53)	0.351	1.11 (0.79–1.57)	0.533	682	202 (30.2)	1.20 (0.97–1.49)	0.351	1.07 (0.83–1.39)	0.591
Delta	902	321 (35.8)	1.49 (1.22–1.81)	<0.001	1.48 (1.21–1.8)	<0.001	1734	531 (30.8)	1.20 (1.62–1.42)	0.026	1.19 (1.01–1.40)	0.038
Years at school												
0 years	234	86 (36.8)	1.00 (ref.)		1.00 (ref.)		438	156 (35.9)	1.00 (ref.)		1.00 (ref.)	
1–5 years	743	218 (29.6)	0.92 (0.67–1.26)	0.598	0.89 (0.64–1.22)	0.465	1810	586 (32.6)	1.12 (0.89–1.41)	0.34	1.13 (0.9–1.44)	0.283
6–11 years	1520	465 (30.8)	1.13 (0.84–1.53)	0.418	1.04 (0.76–1.42)	0.793	1700	462 (27.5)	1.22 (0.96–1.55)	0.109	1.17 (0.92–1.5)	0.207
> 11 years	365	86 (23.7)	0.94 (0.64–1.37)	0.729	0.8 (0.53–1.18)	0.264	509	112 (22.1)	1.14 (0.84–1.56)	0.395	1.08 (0.79–1.5)	0.612
Ethnicity												
Bamar	2094	629 (30.0)	1.00 (ref.)		1.00 (ref.)		3238	629 (30.0)	1.00 (ref.)		1.00 (ref.)	
Other ethnic	768	226 (29.4)	1.02 (0.85–1.24)	0.786	1.03 (0.85–1.24)	0.863	1219	226 (29.4)	1.16 (0.99–1.35)	0.61	1.18 (0.95–1.45)	0.132

^a^SBP ≥ 140 or DBP ≥ 90, or uses antihypertensive medications; ^b^Adjusted for age; ^c^Adjusted for age, rural/urban, region, years at school and ethnicity

In fully adjusted models, both metabolic- and behavioral factors were significantly associated with hypertension (Table 4, Model 2). The OR for hypertension for overweight as compared with normal weight individuals was 2.6 (95 % CI 2.1–3.3) in males and 2.3 (2.0–2.7) in females, while underweight was associated with lower odds. Similarly, increased waist circumference was highly significantly associated with hypertension. Smoking as compared with non-smoking was associated with lower odds for hypertension in women (tobacco: OR = 0.5 (0.4–0.9); smokeless tobacco: OR = 0.77 (0.6–0.9)), while alcohol drinking increased the odds for hypertension among males only (Table 4). Use of sesame oil in cooking as compared with use of peanut oil was associated with lower odds for hypertension in both males (OR = 0.64 (0.5–0.9) and females (OR = 0.75 (0.6–0.9). Vigorous activity at work was also associated with lower odds for hypertension, while there was no association between servings of fruit and vegetables and hypertension.

**Table 4:** Prevalence of hypertension by gender and by selected metabolic- and behaviour determinants and their associations (Odds Ratio (OR)) with hypertension among 15–64 year old citizens of Myanmar

	Male						Female					
		No. (%) with hypertension^a^	Model 1^b^		Model 2^c, d^			No. (%) with hypertension^a^	Model 1^b^		Model 2^c, d^	
	N		OR (95 % CI) ^b^	*P*-value	OR (95 % CI) ^c, d^	*p*-value	N		OR (95 % CI) ^a^	*p*-value	OR (95 % CI) ^c, d^	*P*-value
BMI												
Underweight	569	115 (20.1)	0.66 (0.52–0.84)	0,001	0.63 (0.49–0.82)	0,001	736	118 (16.0)	0.55 (0.52–0.83)	<0.001	0.56 (0.44–0.72)	<0.001
Normal weight	1764	487 (27.6)	1.00 (ref.)		1.00 (ref.)		2344	581 (24.8)	1.00 (ref.)		1.00 (ref.)	
Overweight	479	245 (51.2)	2.55 (2.05–3.16)	<0.001	2.63 (2.08–3.33)	<0.001	1304	611 (46.9)	2.36 (2.03–2.76)	<0.001	2.31 (1.97–2.72)	<0.001
WC												
Normal	2774	819 (29.5)	1.00 (ref.)		1.00 (ref.)		3765	967 (25.7)	1.00 (ref.)		1.00 (ref.)	
Male ≥102 cm, female ≥88 cm	44	30 (68.2)	3.64 (1.89–7.00)	<0.001	3.4 (1.77–6.78)	<0.001	617	344 (55.8)	2.83 (2.36–3.4)	<0.001	2.71 (2.23–3.3)	<0.001
Smoking status												
Never smoker	1302	358 (27.5)	1.00 (ref.)		1.00 (ref.)		3967	1178 (29.7)	1.00 (ref.)		1.00 (ref.)	
Current smoker	1270	389 (30.6)	1.05 (0.88–1.26)	0,58	1.03 (0.86–1.23)	0,719	352	99 (28.2)	0.52 (0.4–0.68)	<0.001	0.53 (0.41–0.69)	<0.001
Former smoker	270	108 (40)	1.25 (0.94–1.67)	0,121	1.2 (0.9–1.6)	0,196	98	39 (39.8)	0.76 (0.5–1.1)	0,206	0.77 (0.5–1.18)	0,23
Smokeless tobacco status												
Never	1268	385 (30.4)	1.00 (ref.)		1.00 (ref.)		3678	1095 (29.7)	1.00 (ref.)		1.00 (ref.)	
Current	1465	427 (29.2)	1.04 (0.88–1.24)	0,638	1.03 (0.84–1.24)	0,67	724	216 (29.8)	0.78 (0.66–0.95)	0,01	0.77 (0.64–0.93)	0,006
Former	109	43 (39.5)	1.45 (0.96–2.21)	0,081	1.41 (0.92–2.15)	0,117	15	6 (40.0)	1.17 (0.39–3.5)	0,769	1.1 (0.37–3.28)	0,863
Daily fruit and veg. use												
≥5 servings	286	77 (26.9)	1.00 (ref.)		1.00 (ref.)		401	109 (27.2)	1.00 (ref.)		1.00 (ref.)	
<5 servings	2320	703 (30.3)	1.10 (0.83–1.47)	0,506	1.06 (0.8–1.42)		3696	1104 (29.9)	1.11 (0.87–1.42)	0,381	1.13 (0.88–1.45)	0,332
Oil use												
Peanut oil	1184	369 (31.2)	1.00 (ref.)		1.00 (ref.)		1873	570 (30.4)	1.00 (ref.)		1.00 (ref.)	
Palm oil	534	179 (33.5)	1.11 (0.89–1.40)	0,357	1.11 (0.87–1.41)	0,406	917	293 (32.0)	1.1 0.92–1.32)	0,266	1.15 (0.95–1.4)	0,159
Mix of peanut and palm oil	326	115 (35.3)	1.09 (0.84–1.43)	0,487	71.08 (0.82–1.43)	0,582	491	160 (32.6)	1.04 (0.83–1.31)	0,693	1.07 (0.85–1.35)	0,543
Sesame oil	532	118 (22.2)	0.59 (0.46–0.76)	<0.001	0.64 (0.49–0.89)	0,001	730	181 (24.8)	0.71 (0.58–0.87)	0,001	0.75 (0.60–0.94)	0,011
Other oil	249	71 (28.5)	0.91 (0.67–1.24)	0,562	0.96 (0.7–1.32)	0,803	377	105 (27.9)	0.87 (0.67–1.12)	0,293	0.89 (0.68–1.16)	0,389
Current alcohol drinkers												
Drinkers	891	314 (35.3)	1.00 (ref.)		1.00 (ref.)		69	20 (29.0)	1.00 (ref.)		1.00 (ref.)	
Non-drinkers	1951	541 (27.7)	0.58 (0.48–0.69)	<0.001	0.57 (0.48–0.69)	<0.001	4348	1296 (29.8)	0.89 (0.52–1.55)	0,707	0.94 (0.53–1.65)	0,817
Vigorous activity at work												
Yes	1167	291 (24.9)	1.00 (ref.)		1.00 (ref.)		664	149 (22.4)	1.00 (ref.)		1.00 (ref.)	
No	1673	563 (33.6)	1.36 (1.14–1.62)	<0.001	1.33 (1.11–1.59)	0,002	3751	1167 (31.1)	1.35 (1.10–1.66)	0,003	1.33 (1.07–1.64)	0,009

^a^ SBP ≥ 140 or DBP ≥ 90, or uses antihypertensive medications; ^b^Adjusted for age; ^c^BMI and WC are adjusted for age, urban/rural, region, years at school, smoking status, smokeless tobacco status, daily fruit and veg use, oil use, current alcohol drinkers and vigorous activity at work; ^d^Smoking status, smokless tobacco status, daily fruit and veg. use, oil use, alcohol and activity at work are adjusted for age, urban/rural, region and years at school

## Discussion

Overall, three out of ten 15–64 year old inhabitants of Myanmar had hypertension, with no difference between the sexes. In fully adjusted logistic regression models, we found increased odds for hypertension by increasing age, and if the participants were exposed to socio-demographic,- metabolic- and behavioral factors like living in urban areas or the delta region, high BMI, high WC, not doing vigorous activity at work, drinking alcohol (males), or non-smoking (females). Using sesame oil in cooking as compared with peanut oil was associated with lower odds for hypertension.

Strengths of our study are the high number of participants and the large age distribution from both urban and rural areas and the different distinct geographical regions across the country. Data was collected using a standardized international accepted STEPS protocol, which may reduce potential information bias. However this type of study, interviewing participants about life-style factors, is prone to recall bias, which may have inflated our association measures. A weakness of the study is that we do not know the extent of non-response, thus, if the non-respondent group differs from those who consent to take part in the study. This possible non-response bias could affect the generalizability of the study, and also the prevalence estimates could be affected. In most studies, people with unhealthy habits tend to be less willing to participate than more healthy individuals [19]. If there is a similar trend in Myanmar, our prevalence estimates are most likely underestimates. Previous studies in western settings, however, have shown that association measures are not much influenced by low response rates [19].

Another source of selection bias is due to selection of participants from each of the households. The participant invited from each household was selected among those who were at home at the time of investigation – not among all those who *belonged* to the household. Males may to a larger degree than females be away from home due to work outside the house, and among elderly, more males than females may be dead. We are unable to estimate the magnitude of the potential bias regarding prevalence estimates, but it is likely that more healthy individuals are working outside the home and, thus, did not have the chance to be included in the study. This may have led to overestimation of the prevalences of NCD risk factors, especially among men. We have adjusted prevalence estimates of hypertension to the world standard population making data available for others to compare, and also conducted all analyses separate for men and women. Although the study claims to be representative for the Union of Myanmar, another limitation was that monks, nuns, and armed forces personnel were not included in the present study. It is estimated that there are between 300 000–500 000 monks in Myanmar, and 375 000 people is serving in the army [20]. It is not known if their health situation differs from the general population and if their exclusion has had any impact on the results.

To conclude regarding potential selection biases: we have probably overestimated prevalence estimates in both male and females, but probably to a larger degree in males, while it is likely that association measures are valid.

Other limitations of the present study is missing information about salt intake, a determinant for hypertension and a potential confounder for the association between adiposity and hypertension. Step 3 of the STEPS protocol, i.e. blood sampling, was not a part of the present study, but blood lipids are most likely intermediate variables and should, thus, not be adjusted for. However, information about salt intake and analyses of lipid profiles should be included in further studies on NCD risk factors in Myanmar. For example, ngapi, which is made by fermenting fish or shrimp and added salt, is a main ingredient in the majority of Burmese cooking. It is a sort of nutritional paste with high salt content and used as a condiment or additive in most dishes. We recommend studying intake of ngapi on hypertension in future surveys in Myanmar. Studies have reported that the salt intake in South-East Asian countries is high [21], and the increase in use of mono-sodium glutamate (MSG) could be a major contributor to the burden of hypertension. MSG and salt intake are highly associated with high blood pressure [7, 22–24].

A recent published systematic review about prevalence and risk factors of hypertension in Myanmar [10] report a lower over-all prevalence point estimate of hypertension (22 %) as compared with the present study, but its 95 % confidence interval (14–31 %) is overlapping the point estimate of the present study. There are some limitations of the systematic review. Out of the seven included studies, only one is recently conducted. The others are from the start of the 2000s, and one dates back to 1992. Thus, they used relatively old information that does not necessarily describe the current situation in Myanmar. At the same time, we acknowledge that our data was collected in 2009, and may not represent the current situation. We believe the burden of NCD risk factors is higher today. The review [10] has in addition included the same study two times, the Myanmar STEPS Survey from 2003 to 2004 [25] from Yangon division. Furthermore, it is likely that they have not had access to original data, thus no ability to consider for example outliers/odd values and exclusion of pregnant women, although 110 pregnant women will only have marginal impact on hypertension estimates.

In a STEPS study from Yangon Region in 2003–2004 among 20 years and older male and females, the prevalence of hypertension was 33.8 % [25], slightly higher than in the present study. However, both studies found no sex differences in prevalence rates. The higher rates in the Yangon study, although it was conducted 5–6 years earlier, could be because they included older participants than in the present study. When comparing prevalence estimates age adjusted to the WHO standard population, the prevalence of hypertension was slightly higher in the present nationwide study (29.9 %) compared with the Yangon study (27.2 %). Additionally, Yangon Region is among the most developed and urbanized parts of the country, and it’s not surprising that risk factors linked to urbanization are more unfavorable than found in our nationwide study. Thus, due to geographical differences between the two studies, we are not able to conclude on trends in hypertension from 2003/2004 to 2009. When comparing studies from neighboring countries, the prevalence of hypertension in Myanmar is at the same level as in India (29.8 %; 95 % CI 26.7–33.0) [26], but higher than reported from Thailand (21.5 %, 19.3–23.8) [27], Bangladesh (13.5 %; 12.7–14.3) [28] and China (26.6 %) [29]. We report higher hypertension estimates in Myanmar than the overall worldwide prevalence, but lower than established market economies like Spain, England, Germany and Sweden [30]. Although Myanmar has a market oriented economy, the lower prevalence than in other marked economies may be explained with a unfinished transformation [31] and that the economy benefits the population unevenly [5], in addition to health system factors and individual factors.

Our analyzes of associations between hypertension and socio-demographic,- metabolic- and behavioral factors, show a similar pattern described in several other studies [32, 33]. The epidemiologic transition in low income countries is often explained by economic development which leads to urbanization with a more sedentary life style, changes in dietary habits, more smoking and social stress [34]. It is likely that Myanmar is undergoing a similar development.

In the Systematic review from Myanmar [10], a positive association between smoking and hypertension was demonstrated. Surprisingly, in the present study smoking was not associated with hypertension in men, and showed an inverse relationship among women, partly supported with findings from the STEPS study from Yangon Region in 2003-2004 [25]. In that study, no association between smoking and hypertension was found, but separate figures for male and female were not reported. The present findings of a negative association in female and no association in male call for further investigation. An association between alcohol drinking and hypertension was found in men only, while in the Yangon Region study, an association was reported with male and females combined [25]. The prevalence of alcohol drinking among women in Myanmar is low, due to culture, tradition and the Buddhist religion, and calls for sex-stratified analyses. The same applies to analyses of smoking.

Our findings of overweight and low physical activity as determinants of hypertension support previous studies from Myanmar [25] and further support a general view that adiposity is a major determinant of hypertension [35]. A recent study of relatively lean rural adults in southwest of China reported that general adiposity in terms of higher BMI is more strongly associated with hypertension than waist circumference [36]. In the present study we found an opposite tendency, that waist circumference is more strongly associated with hypertension than BMI above 25 kg/m^2^. The failure of finding an association between hypertension and low levels of fruit and vegetables in the present study and the scarcity of studies from low income countries calls for more research. Our finding of lower odds for hypertension among those who report use of sesame oil for cooking, as compared with peanut oil supports previous studies of the beneficial effect of sesame oil in reduction of blood pressure in hypertensive patients [37].

Given a continuous economic development in Myanmar, and if preventive policies for NCDs are not put in place, we may expect a similar epidemiologic transition in Myanmar as in other Asian countries, like for example the well documented NCD trends in China [38]. The development seen in Singapore during the past several decades could also indicate future prospects of Myanmar: a rapid urbanization and economic development, with a demographic transition concomitant with an epidemiological transition, leading to an increase in cardio vascular diseases and their associated factors [3]. There is available evidence for health promoting interventions in reducing NCD risk in low income setting [39], which could be considered in plans for NCD policies in Myanmar.

## Conclusions

We conclude that the prevalence of hypertension among 15–64 year old citizens of Myanmar is high and associated with socio-demographic,- metabolic- and behavioural factors. However, prevalence estimates may have been overestimated due to several sources of potential selection biases. Future studies on NCD risk factors should pay attention to sampling procedures to reduce biases. The large group of military people, monks and nuns should be included in new studies in order to make a sample representative for the population of Myanmar. Alcohol intake, smoking and nutritional factors including salt intake, should be investigated in more detail.

### Public health implications

The prevalences of hypertension and associated factors are high and probably increasing. We recommend studies on effectiveness on interventions and immediate implementation of policies to reduce the burden of NCD risk based on programs proven effective in similar settings [39].

## Abbreviations

BMI, body mass index; CI, confidence interval; DAG, directed acyclic graphs; MSG, mono-sodium glutamate; NCDs, non-communicable diseases; OR, odds ratio; PPS, probability proportionate to population size; SSU, secondary sampling unit; STEPS, STEPwise approach to surveillance; WC, waist circumference; WHO, World Health Organization
